# Transient-Evoked Otoacoustic Emissions May Reveal Damage to Outer Hair Cells Caused by Exposure to Recreational Noise: A Narrative Review

**DOI:** 10.3390/medicina61091538

**Published:** 2025-08-27

**Authors:** Ziqi Zhou, Xingqian Shen, Linlin Wang, Xiaoye Chen, Ting Li, Bo Liu, Hongjun Xiao

**Affiliations:** 1 Department of Otorhinolaryngology-Head and Neck Surgery, ENT Institute, Union Hospital, Tongji Medical College, Huazhong University of Science and Technology, Wuhan 430022, China; 2 Clinical Medical Research Center of Deafness and Vertigo in Hubei Province, Wuhan 430022, China

**Keywords:** recreational noise-induced hearing loss, transient-evoked otoacoustic emission, outer hair cells, early screening

## Abstract

Recreational noise-induced hearing loss (RNIHL) is a significant factor contributing to hearing loss in young people. Its process is irreversible, and early symptoms are hidden. Therefore, early identification is of great significance. Otoacoustic emissions (OAEs) are commonly used to detect the function of outer hair cells. It is widely used for early diagnosis of occupational noise-induced hearing loss, but it remains underutilized in RNIHL detection. In order to explore the characteristics of RNIHL and the early detection value of different types of OAEs, this study reviewed the detection results of OAEs in previous studies on noise-induced hearing loss and analyzed the differences and causes among the studies. The study found that, through the detection of distortion product otoacoustic emissions (DPOAEs), there were differences in the sensitive frequency bands of outer hair cell damage between recreational noise and occupational noise, particularly in the high-frequency region. Transient-evoked otoacoustic emissions (TEOAEs) can fully reflect the damage to cochlear outer hair cells caused by RNIHL. This study indicates that OAEs, particularly TEOAEs, can serve as a highly sensitive and objective detection tool for RNIHL, whereas DPOAEs are more appropriate for the early screening of occupational noise-induced hearing loss.

## 1. Introduction

Noise-induced hearing loss (NIHL) is a type of auditory system impairment produced by continuous and excessive noise exposure [1]. According to the reports, about 1.3 billion people suffer from hearing loss due to noise exposure [2]. Although occupational factors are the primary causes of noise-induced hearing loss, the damage from recreational noise cannot be ignored: studies indicate that high-risk cumulative leisure noise in people aged 50–79 years old is associated with hearing abnormalities, and increased recreational noise exposure among adolescents with insufficient use of hearing protection measures poses risks of hearing damage such as noise-induced threshold shifts [3,4]. Recreational noise-induced hearing loss (RNIHL) is associated with various hearing-related symptoms (such as pain in the ear, tinnitus, and hyperacusis) and non-auditory systems (such as sleep disturbances, anxiety, depression, and cognitive dysfunction). Many patients seeking medical aid may experience the above-mentioned discomfort and exposure to recreational noise, but conventional pure-tone audiometry results are normal [5]. The lack of more sensitive and effective early detection methods for RNIHL has led many young people to overlook the hazards of recreational noise exposure and rarely use hearing protection devices even in strong noise environments [6]. This further exacerbates the hearing loss of these patients, resulting in a greater socio-economic burden. Therefore, detection methods that are highly sensitive and can be used for early screening are extremely valuable for RNIHL.

The main pathological manifestation of NIHL in the inner ear is damage and loss of hair cells, with outer hair cells (OHCs) being more commonly and earlier damaged due to their distinctive physiological characteristics [7]. OHCs have the functions of mechano-electrical transduction and active amplification of sound signals. This special physiological function makes them more vulnerable to noise stimuli [8]. Once the noise intensity exceeds their tolerance threshold, OHCs will exhibit structural and functional abnormalities earlier. Otoacoustic emissions (OAEs) are forms of acoustic energy generated by the activity of OHCs in the cochlea, which can be collected and recorded by a microphone fitted into the ear canal, reflecting the integrity of the cochlear OHCs [9]. OAEs are quite sensitive to the damage of OHCs. When OHCs are damaged, a decrease in the amplitudes and even a failure to elicit OAEs can be observed. Therefore, OAEs may be a promising tool to detect RNIHL [6].

Previous studies explored the application value of various types of OAEs in detecting occupational noise-induced OHCs impairments and a few in recreational noise-induced OHCs impairments, and the results were inconsistent among different OAEs. Biassoni et al. used transient-evoked otoacoustic emissions (TEOAEs) to monitor teenagers’ hearing over 3 years and found that the amplitude of TEOAEs decreased with the increase of the hearing threshold [10]. Paping et al. examined distortion product-evoked otoacoustic emissions (DPOAEs) in observing recreational noise-induced hearing loss in adolescents, and the findings indicated that DPOAEs could screen for hearing loss at early stages [11]. Laffoon et al. found that DPOAEs would be useful for early evidence of cochlear damage from recreational firearm impulse noise in youth [12]. Furthermore, Ellison et al. compared the performance of stimulus-frequency otoacoustic emissions (SFOAEs) with DPOAEs and TEOAEs, and found that SFOAEs were also capable of identifying the presence of hearing loss, with better performance in predicting auditory status at 0.5 kHz than the latter two [13]. However, the value of various types of OAEs in detecting hearing loss caused by different noises is still unclear. Especially for the detection of RNIHL, the inconsistent populations and indicators used in various studies have led to confusing results.

Given the increasingly severe status of RNIHL among young people and the critical significance of early detection in preventing and treating this disease, we aim to review previous studies and compare the differences in OAEs between RNIHL and occupational NIHL, clarify the clinical application value of different types of OAEs in the detection of RNIHL, provide more reliable and effective detection methods for early screening and intervention of this disease, and help reduce the harm of RNIHL.

## 2. Principles of Otoacoustic Emission

OAEs are by-products of the “cochlear amplifier”, which is essentially the form of sound energy generated by the activity of OHCs in the cochlea. The special electro-mechanical characteristics of the OHCs cause the cell to contract or elongate when it is depolarized or hyperpolarized by sound-induced stimulation. This change in length exerts an additional force on the basilar membrane, enhancing its vibration, a process known as the “cochlear amplification” function of OHCs. It also greatly improves the cochlea’s capacity to detect and distinguish faint sounds. A part of the mechanical energy is retropropagated as sound waves during this process, moving from the cochlea to the external auditory canal via the middle ear. And subsequently captured by a microphone placed in the ear canal. The recorded sound waves are amplified and sharpened in different frequency bands according to the stimulus frequency. When noise damages the OHCs, their electro-mechanical properties are changed, impairing the OHC’s amplification capability and decreasing the amount of OAEs energy produced, which is manifested as a reduction in amplitude or disappearance of the OAEs [14].

## 3. Recreational Noise and Occupational Noise

**Figure 1:**
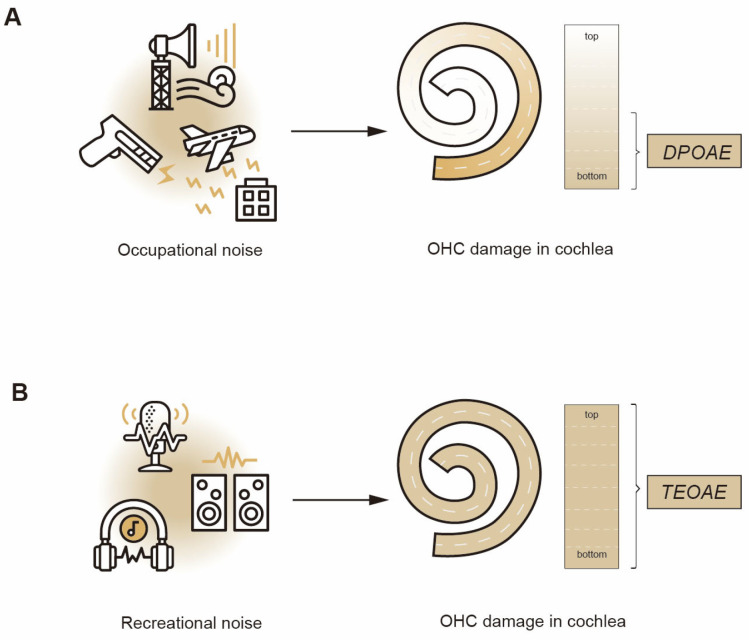
The damage patterns and characteristic detection methods of OHCs caused by different noises. The dark areas indicate the areas of outer cell damage. (**A**) The damage patterns and characteristic detection methods of OHCs caused by occupational noise. (**B**) The damage patterns and characteristic detection methods of OHCs caused by recreational noise. OHC: outer hair cell; DPOAE: distortion product-evoked otoacoustic emission; TEOAE: transient-evoked otoacoustic emission.

Occupational noise exposure predominantly affects industrial workers (e.g., those in shipyards, cotton mills, steel mills, and the construction industry), military personnel, and pilots. Daily noise exposure level in industrial settings is between 85 and 120 dB (sound pressure level, SPL) [15,16]. And pilots experience noise exposure at approximately 100 dB, military personnel face high-intensity impulse noise with peak levels exceeding 150 dB from firearms and explosives. In contrast to occupational noise exposure, recreational noise exposure primarily affects adolescents and young adults. It may be related to loud recreational venues (e.g., pubs and concerts with sustained levels > 100 dB), sporting events (80–120 dB) [17], and personal music players (PMPs) at volumes reaching 105 dB. And in some cases (such as the sound of band drums), it can also generate impulse noise exceeding 140 dB [18]. As to the sound level, recreational noise may have greater noise exposure. Martínez-Wbaldo et al. investigated hearing loss in high school teenagers and found that youngsters’ recreational noise exposure often exceeds the recommended safety limits, with the recommendation that noise exposure should not be more than 8 h at 90 dB and should not be more than 4 hours at 93 dB [18]. In addition, when comparing the noise spectrum, the recreational noise spectrum may exhibit broader frequency bandwidth and greater variability. Therefore, as shown in Figure 1, compared to the characteristic OHC damage at the bottom of the cochlea caused by occupational noise, recreational noise may affect the OHCs of the entire cochlea.

## 4. Classification of OAEs and Application to NIHL

Depending on whether induced by outside stimuli, otoacoustic emissions are classified into two types: spontaneous otoacoustic emissions and evoked otoacoustic emissions. Spontaneous otoacoustic emissions are weak and sensitive to factors as age, gender, inner ear pathology, and the sensitivity of the test equipment. As a result, there are significant individual differences among normal subjects, and the incidence of spontaneous otoacoustic emission is low, only about 30~40% [19], so their clinical application is restricted. Evoked otoacoustic emissions are further classified into three categories based on the type of stimuli: DPOAEs, SFOAEs, and TEOAEs. The development of DPOAEs, TEOAEs, and SFOAEs research and their use in RNIHL are the main topics of this section.

### 4.1. Distortion Product Otoacoustic Emissions (DPOAE)

DPOAEs is based on the nonlinear characteristics of cochlear outer hair cells, which produce distorted products when two different frequency pure tones are simultaneously transmitted. It is generally believed that it can better reflect the function of outer hair cells around the bottom of the cochlea and the cochlea’s ability to respond to high-frequency sounds [20,21].

Analyses of occupational NIHL demonstrate that audiometric damage initially occurs in the high-frequency range [22], and Figure 1A shows the main areas of OHCs damage. Animal studies have further shown that OHCs exhibit greater susceptibility and vulnerability to high-frequency stimulation [14]. Previous studies have explored the detection efficacy of DPOAEs in populations exposed to noise, and we have summarized these studies in Table 1. Shupak et al. observed a reduction in DPOAE amplitudes through a two-year follow-up study of individuals exposed to noise, with obvious declines at frequencies around 4 kHz, 5 kHz, and 6 kHz [23]. Seixas et al. used pure-tone audiometry (PTA) and DPOAEs to investigate construction workers’ hearing conditions, and observed a significant correlation between cumulative noise exposure and DPOAEs amplitude declines at 4 kHz and 6 kHz [24]. A cross-sectional study by Wei et al. found that DPOAE amplitudes in young factory workers decreased at 3, 4, and 5 kHz [25]. These findings indicate that DPOAEs monitoring for occupational NIHL primarily is at the 4–6 kHz frequency range. As for RNIHL, the results of DPOAEs seem to behave differently. Le Prell et al. evaluated hearing performance from 74 adult participants who were exposed to recreational sound exposure, and perceived that with the thresholds increasing at 6 kHz and 8 kHz, the DPOAEs amplitudes decreased [26]. When Narahari et al. assessed the immediate and short-term effects of PMS usage on hearing, they found a significant reduction in DPOAEs amplitude in the frequency range of 9 to 12 kHz [27]. Laffoon et al. investigated cochlear damage from recreational firearm noise and identified that DPOAEs was particularly sensitive at 8 and 10 kHz [12]. Such results suggest that RNIHL may affect OHCs at a higher frequency than occupational noise. That may be related to the fact that recreational sound involves more complex high-frequency components.

In addition, although the outer hair cells that respond to high-frequency sounds have a higher susceptibility to noise, the impact of low-frequency noise is also receiving increasing attention [8,28,29], which may affect auditory function by damaging the parietal outer hair cells. However, recreational noise has poor frequency specificity and may contain a large amount of low-frequency noise or even infrasound. Therefore, DPOAEs may not fully reflect the outer hair cell damage of RNIHL, and DPOAEs are more suitable for early screening of occupational NIHL rather than RNIHL.

**Table 1:** Summary of studies on DPOAEs in NIHL.

Author	Jobs	Age	Indicators	Frequency Range of Measurement	Results
Gopal et al., 2019 [30]	adults	18–31	amplitudes	499, 1003, 1409, 2000, 2822, 3991, and 5649 Hz	DPOAEs amplitudes reduced to 2 kHz, 3 kHz
Narahari et al., 2017 [27]	students	17–22	amplitudes	2–12 kHz	DPOAEs amplitudes reduced to 9–12 kHz
Seixas et al., 2005 [24]	construction industry workers	not mentioned	amplitudes	500, 1000, 2000, 3000, 4000, 6000, and 8000 Hz	DPOAEs changed to 4 kHz
Wei et al., 2025 [25]	workers	<42	amplitudes	0.5, 1, 1.5, 2, 3, 4, 5, 6, 7, 8, 9, and 10 kHz	DPOAEs amplitudes reduced to 3, 4, 5 kHz
Le Prell et al., 2018 [26]	colleges	18–27	amplitudes	2210, 2782, 3506, 4416, 5565, 7013, and 8837 Hz	DPOAEs amplitudes reduced
Pawlaczyk-Łuszczyńska et al., 2021 [31]	students	19–32	amplitudes, SNR	750–9680 Hz	DPOAEs amplitudes reduced to 6 kHz, and near 8 kHz
Plinkert et al., 1999 [32]	soldiers	18–35	amplitudes	1–6 kHz	DPOAEs SNR reduced to 984 Hz, 6 kHz, and near 8 kHz
Shupak et al., 2007 [23]	Ship workers	18–20	amplitudes	928–12,012 Hz	DPOAEs alterations were not significant
Dudarewicz et al., 2022 [33]	Ultrasonic device operators	43.1 ± 10.8	amplitudes, reproducibility, SNR	1.5–10 kHz	DPOAEs cannot be used as an objective measure of pure-tone thresholds in early NIHL

DPOAE: distortion product-evoked otoacoustic emission; NIHL: noise-induced hearing loss; SNR: signal-to-noise Ratio.

### 4.2. Transient-Evoked Otoacoustic Emissions (TEOAEs)

TEOAEs are an acoustic signal recorded in the external auditory canal following transient stimulation of the cochlea. This transient sound is usually a click or chirp tone burst. Click is a wide-band stimulus that quickly excites the whole cochlea, reflecting the overall functional status of all OHCs across the range of 0.5–4 kHz. A tone burst is a sinusoidal signal. In contrast to the click sound, this single-frequency sound makes itself frequency-specific. This characteristic allows it to selectively stimulate OHCs in specific regions of the cochlea. As a result, tone-burst sound is particularly helpful for more precisely assessing the functional status of OHCs at specific locations within the cochlea.

As shown in Table 2, it was found that the detection frequency range was different between TEOAEs and DPOAEs. Budak et al. used TEOAEs to monitor the OHCs function of 31 noise-exposed carpenters, and the result showed that the TEOAEs were significantly decreased at 2 kHz and 2.8 kHz [34]. In a cross-sectional study of 31 hot air balloon pilots, Sahin Ceylan et al. observed a negative correlation between TEOAE amplitude of 4 kHz and the duration of noise exposure [35]. The above results indicate that although TEOAEs are not as sensitive as DPOAEs for detecting hearing loss above 6 kHz, TEOAEs have the same application value as DPOAEs in detecting noise hearing loss around 2–4 kHz. Similar results were observed when using TEOAEs to assess RNIHL. When Nambiar et al. proposed to profile audiological characteristics and document hearing-related symptoms in artists, they found TEOAEs absent at 3 and 4 kHz [36]. Rosanowski et al. investigated whether TEOAEs can be used to examine OHC damage caused by leisure noise, and observed that the TEOAE level and reproducibility decreased significantly at 3 kHz [37]. Furthermore, another outcome was detected when analyzing TEOAEs using the parameter SNR. Pawlaczyk-Łuszczyńska et al. found that music students had a lower SNR of TEOAEs in the 1 kHz band [31]. This demonstrated that TEOAEs may have the potency to predict OHC damage in the <2 kHz frequency range.

The above results suggest that recreational noise may damage a wider range of OHCs than occupational noise, as shown in Figure 1. And DPOAEs are more suitable for detecting OHC loss at >6 kHz, whereas TEOAEs are more appropriate for finding OHC injury at <4 kHz. However, which one is more perfect for the detection of RNIHL has not been pointed out in previous studies. Plinkert et al. tested different audiological methods (PTA, extended high-frequency audiometry, TEOAEs, DPOAEs, etc.) to detect a high noise susceptibility and suggested that TEOAEs provided a more sensitive and objective method for identifying subtle pathological changes caused by noise exposure [32]. Dudarewicz et al. investigated the hearing status of 148 operators working with ultrasound equipment, and although both TEOAEs and DPOAEs reflected hearing loss, DPOAEs were better at specifically identifying NIHL [33]. Shupak et al. observed the changes in TEOAEs, DPOAEs, and PTA during the first 2 years of occupational noise exposure. The results reflected that abnormal TEOAE parameters after the first year of noise exposure had a high sensitivity for predicting NIHL after 2 years [23]. The discrepancies in these findings may be related to the different parameters and subjects’ heterogeneity. For example, hormonal factors may be one of the reasons. Males show a higher DPOAEs absence rate, so if there is an uneven distribution of the gender in experimental and control groups, it may produce significant variability [4]. But the most important reason is the different sensitivity of TEOAEs and DPOAEs to OHCs. As shown in Figure 1B, TEOAEs originate from the entire length of the basilar membrane; even a narrow frequency band can change the TEOAEs waveform [38,39]. Nevertheless, DPOAEs only reflect the OHCs at a specific stimulus f2 frequency field [40].

Thus, compared to DPOAEs, the ability of TEOAEs to predict RNIHL has greater potential. This superior capacity has also been demonstrated by Miller’s study [41].

**Table 2:** Summary of studies on TEOAEs in NIHL.

Author	Jobs	Age	Indicators	Frequency Range of Measurement	Results
Budak et al., 2021 [34]	carpenters	25–60	amplitudes	1000, 1400, 2000, 2800, and 4000 Hz	TEOAEs amplitudes reduced to 2 kHz and 2.8 kHz
Nambiar et al., 2024 [36]	artists	>18	present or absent	500–4 kHz	TEOAEs absent at 3 kHz and 4 kHz
Sahin Ceylan et al., 2023 [35]	pilots	not mentioned	amplitudes	1–4 kHz	TEOAE amplitudes reduced at 4 kHz
Rosanowski et al., 2006 [37]	students	20–25	reproducibility, level, SNR	0.5–4 kHz	The level and reproducibility decreased significantly at 3 kHz
Pawlaczyk-Łuszczyńska et al., 2021 [31]	students	19–32	amplitudes, SNR	1, 1.5, 2, 3, 4 kHz	TEOAEs SNR reduced at 1 kHz
Plinkert et al., 1999 [32]	soldiers	18–35	amplitudes	1–4 kHz	TEOAEs amplitudes altered more sensitively
Shupak et al., 2007 [23]	Ship workers	18–20	amplitudes	1, 1.5, 2, 3, and 4 kHz	TEOAEs changes more sensitively
Dudarewicz et al., 2022 [33]	Ultrasonic device operators	43.1 ± 10.8	amplitudes, reproducibility, SNR	1, 1.5, 2, 3, and 4 kHz	TEOAEs amplitudes fell at all frequencies

TEOAE: transient-evoked otoacoustic emission; NIHL: noise-induced hearing loss; SNR: signal-to-noise Ratio.

### 4.3. Stimulated Frequency Otoacoustic Emissions (SFOAE)

SFOAEs are elicited by a single continuous pure-tone signal and are primarily believed to result from linear coherent reflection [42]. SFOAEs have a unique advantage in evaluating OHC frequency specificity [43]. Dewey et al. studied SFOAEs in more than the standard hearing range (>8 kHz) and found that it could still be created, albeit with a substantial decrease in amplitude [44]. This suggests that SFOAEs may compensate for the limitations of TEOAEs and DPOAEs. However, there are fewer clinical studies on SFOAEs, mainly because it is more difficult to detect than other types of otoacoustics. It is hoped that this difficulty will not be an obstacle for us, though, with the continuous development of acoustic testing techniques.

## 5. OAEs Compared to Other Methods for Detecting RNIHL

To comprehensively compare the performance of other hearing detection methods against OAEs in NIHL—with a particular focus on their applicability in early screening and diagnosis—this section reviews the characteristics and efficacy of pure-tone audiometry (PTA), auditory brainstem response (ABR), and electrocochleography in direct contrast to OAEs. For a clear visual synthesis of key findings from relevant comparative studies, we have summarized these results in Table 3.

PTA is a traditional tool to detect occupational NIHL. However, because it is a subjective audiological test, subjects who are unable to fully understand the task or cooperate may affect its accuracy [45]. In addition, it is insensitive to small pathological changes. Extensive studies have demonstrated that TEOAEs and DPOAEs exhibit higher sensitivity than PTA in the early detection of NIHL [36,46,47]. Helleman et al. conducted a 17-month longitudinal study of 233 printing industry workers and learned that DPOAE amplitudes declined significantly at high frequencies, with greater sensitivity compared to PTA [46]. Capozzella et al. simultaneously used PTA and DPOAEs to assess hearing loss in workers, revealing that DPOAEs are superior to PTA for early detection of hearing damage [47]. Meng et al. used DPOAEs, TEOAEs, and extended high-frequency audiometry to observe hearing loss in 86 young adults and found that TEOAEs may be the earliest indicator of minor lesions in NIHL [7]. Konopka et al. compared hearing outcomes between males exposed to impulse noise and non-exposed controls by using TEOAEs and PTA. They reported that TEOAEs are more sensitive than PTA in identifying NIHL [48].

ABR is a series of short-latency auditory evoked responses to transient acoustic stimulus signals recorded from scalp electrodes in the auditory nerve and brainstem pathways. These response waves usually appear within 10 ms of stimulation and are represented sequentially by Roman numerals (I to VII), with I, III, and V commonly used in clinical practice. It has been shown that NIHL can affect the ABR waveform amplitude, but the use of the ABR to detect early hearing loss is not stable. An animal study in mice by Kokash et al. found that the amplitude of the ABR was significantly altered during the first 30 days of noise exposure, and there was no significant change in ABR amplitude at 45 days, which may stem from a temporary noise-induced shift in hearing thresholds [49]. Fraenkel et al. compared the sensitivity of ABR, TEOAEs, and DPOAEs to the NIHL and showed that TEOAEs and DPOAEs were superior to ABR in detecting mild noise hearing loss [50]. Some research suggested that ABR Wave I could detect cochlear synaptopathy, which is a precursor to NIHL; however, current findings exhibit significant heterogeneity [51].

Electrocochleography is an electrophysiological technique that records the electrical activity of the cochlea and primary auditory nerve fibers following acoustic stimulation. ECochG typically consists of three major waveform components: the cochlear microphonic potential (CM), the summating potential, and the compound action potential of the auditory nerve. CM is generated by outer hair cells and a small part of inner hair cells. Its absence also represents the impaired hair cell function. However, we did not find research about the relationship between RNIHL and CM or between occupational hearing loss and CM. Wang et al. found the elicited rate of CM was obviously higher than that of DPOAEs in the same patient with profound sensorineural deafness [52]. Starr et al. used CM and TEOAEs to define both auditory nerve and cochlear receptor functions in auditory neuropathy patients and found their CM amplitudes elevated and TEOAEs absent [53]. The above results illustrate that CM may have the potential value to find RNIHL.

**Table 3:** Summary of studies on OAEs compared to other methods for detecting NIHL.

Author	OAEs Type	Other Methods	Subjects	Indicators	Results
Helleman et al., 2010 [46]	DPOAEs and TEOAEs	PTA	workers	amplitudes	DPOAEs and TEOAEs show a decline in a larger frequency region than PTA
Santaolalla Montoya et al., 2008 [54]	DPOAEs and TEOAEs	PTA	young adults exposed to MP3 player noise	present and amplitudes	TEOAEs and DPOAEs can detect cochlear impairment before the impairment becomes clinically apparent
Capozzella at al., 2015 [47]	DPOAEs	PTA	workers	hearing loss detection rate	The higher effectiveness of DPOAEs in making an early diagnosis of hearing loss
Seixas et al., 2005 [24]	DPOAEs	PTA	construction industry workers	amplitudes	DPOAEs is more sensitive to early changes than standard PTA
Nambiar et al., 2024 [36]	TEOAEs	Extended high-frequency audiometry	artists	present	TEOAEs absent at 3 kHz and 4 kHz Extended high-frequency audiometry showed no response at the majority of thresholds
Konopka et al., 2007 [48]	TEOAEs	PTA	workers	amplitudes	The reduction of TEOAEs was incommensurably greater than the changes in PTA
Rezaee et al., 2012 [55]	TEOAEs	PTA	soldiers	amplitudes	TEOAEs is more sensitive than PTA in detecting early hearing loss after military shooting exercises
Wang et al., 2021 [56]	DPOAEs and TEOAEs	ABR	adults	amplitudes	ABR wave I amplitudes decreased at 1 day post-acute recreational noise exposure at high intensity
Fraenkel et al., 2003 [50]	TEOAEs	ABR	mice	amplitudes	TEOAEs were more sensitive in detecting changes
Wang et al., 2010 [52]	DPOAEs	CM	sensorineural deafness	elicited rate	The elicited rate of CM was higher than DPOAEs
Starr et al., 2001 [53]	TEOAEs	CM	auditory neuropathy patients	amplitudes, latencies	CM amplitudes elevated and TEOAEs are absent

OAE: otoacoustic emission; TEOAE: transient-evoked otoacoustic emission; DPOAE: distortion product-evoked otoacoustic emission; PTA: pure-tone audiometry; ABR: Auditory Brainstem Response; CM: cochlear microphonic potential.

## 6. More Applications of OAEs in NIHL

Regarding the exploration of cochlear mechanisms and clinical early identification of NIHL using OAEs, some studies have discussed certain other applications beyond traditional methods. Zimatore et al. proposed a new parameter from TEOAEs to distinguish normal hearing from hearing impairment in the early stages [57]. This new parameter is analyzed through a multivariate technique and called RAD2D. Through their work, they found this new parameter could identify hearing impairments much earlier than the conventional TEOAEs pass/fail rate [57]. Madzivhandila et al. combined TEOAEs with a mobile phone, which included a Codec device, a probe, and a microphone [58]. This device has been proven to detect hearing loss in infants. If it is widely used in recreational noise exposure among adults, it could facilitate the early identification of hearing loss, enabling timely scientific management and intervention, thereby reducing the social and economic burdens associated with hearing impairment [58]. These innovative techniques further enhance the clinical utility of TEOAEs in RNIHL and reveal possibilities for the early prevention and management of it.

Tinnitus is the perception of sound unrelated to an external or internal source and is often considered to be one of the precursor symptoms of RNIHL [36,59]. Previous studies have established a positive association between noise exposure and the incidence rate and severity of tinnitus [60]. Furthermore, studies found that people who are regularly exposed to recreational noise show a higher prevalence of tinnitus [61,62]. In addition, Vardonikolaki et al. in their study of musicians’ hearing noticed that the DPOAEs and TEOAEs SNR decreased among the musicians with tinnitus, despite having normal audiograms [63]. Fernandes et al. divided 40 subjects with normal hearing into two groups: with and without tinnitus and observed that the tinnitus group had a lower TEOAE amplitude [64]. In contrast, the without-tinnitus group showed no significant changes [64]. Paglialonga et al. showed that the amplitudes of TEOAEs in some normal hearing tinnitus patients reduced in the frequency range of around 1.5 kHz to 8 kHz [65]. These studies revealed that the changes in TEOAEs and DPOAEs would be an indicator of RNIHL for tinnitus with normal hearing.

The medial olivocochlear reflex (MOCR) is a self-protective mechanism of the organism that reduces bilateral OHC gain to decrease high-intensity noise-induced damage to the auditory system. The function of the MOCR can be estimated by measuring the contralateral suppression of DPOAEs and TEOAEs [66]. MOCR strength was found to be related to speech perception in noise conditions, which could be an early manifestation of NIHL. Megha et al. compared the TEOAEs contralateral suppression between noise-exposed and control groups and found weaker suppression in the noise-exposed group [67]. Maison et al. observed that the contralateral suppression of DPOAEs was negatively correlated with the hearing degree of NIHL [68]. Elangovan et al. assessed the role of MOCR in leisure noise exposure and found that it may be an effective tool for detecting the early stages of RNIHL [69]. All in all, these findings suggest that the function of the contralateral suppression of TEOAEs and DPOAEs may be a new method to detect RNIHL at the early stage.

## 7. Strengths and Limitations

This study comprehensively reviewed previous research, elucidated the characteristics of NIHL caused by different types of noise, and analyzed the early detection value of different types of OAEs. We found that DPOAEs may be more suitable for the early identification of occupational NIHL, as the two have high consistency and uniformity in frequency. However, for complex and variable RNIHL, TEOAEs should be used as an early screening tool, as they can detect damage to the outer hair cells along the entire length of the cochlea. This finding is of great significance, especially for RNIHL, which is easily overlooked and underrecognized. These patients are often young and do not have the relevant factors that lead to age-related hearing loss. Early identification and intervention of the damage caused by recreational noise to their auditory function will greatly delay the progression of their hearing loss.

Undoubtedly, this study also has certain limitations. Due to the inconsistent inclusion of subjects in previous studies on RNIHL, we were unable to conduct a systematic evaluation across studies. This is determined by the variability and diversity of recreational noise. This further demonstrates the value of TEOAEs detection in RNIHL. Further large-scale database analysis may be required in the future.

## 8. Conclusions

Nowadays, more and more young people suffer from RNIHL. However, there is no standard criterion to diagnose this problem. From this review, OAEs may be a highly sensitive and objective test for RNIHL, especially TEOAEs. And DPOAEs may be more suitable for the early detection of occupational NIHL.

## Data Availability

No new data were created or analyzed in this study. Data sharing is not applicable to this article.

## Abbreviations

The following abbreviations are used in this manuscript:

RNIHL	recreational noise-induced hearing loss
OAE	otoacoustic emission
DPOAE	distortion product-evoked otoacoustic emission
TEOAE	transient-evoked otoacoustic emission
NIHL	noise-induced hearing loss
OHCs	outer hair cells
SFOAE	simulated frequency otoacoustic emission
MOCR	medial olivocochlear reflex
ABR	auditory brainstem response
PTA	pure-tone audiometry
CM	cochlear microphonic potential
